# Correction: Mamardashvili et al. New Polyporphyrin Arrays with Controlled Fluorescence Obtained by Diaxial Sn(IV)-Porphyrin Phenolates Chelation with Cu^2+^ Cation. *Polymers* 2021, *13*, 829

**DOI:** 10.3390/polym16050618

**Published:** 2024-02-23

**Authors:** Galina M. Mamardashvili, Dmitriy A. Lazovskiy, Ilya A. Khodov, Artem E. Efimov, Nugzar Z. Mamardashvili

**Affiliations:** G.A. Krestov Institute of Solution Chemistry of Russian Academy of Sciences, Akademicheskaya st. 1, 153045 Ivanovo, Russia; gmm@isc-ras.ru (G.M.M.); lazolvo@mail.ru (D.A.L.); ilya.khodov@gmail.com (I.A.K.); artem.efimov.1995@list.ru (A.E.E.)

## Error in Figures

In the original publication [1], the authors identified a discrepancy in Figure 6 and Figure 9. The corrected figures appear below. The original publication has been updated with a minor correction to the correspondence contact information. The authors state that the scientific conclusions are unaffected. This correction was approved by the Academic Editor. The original publication has also been updated.

## Figures and Tables

**Figure 6:**
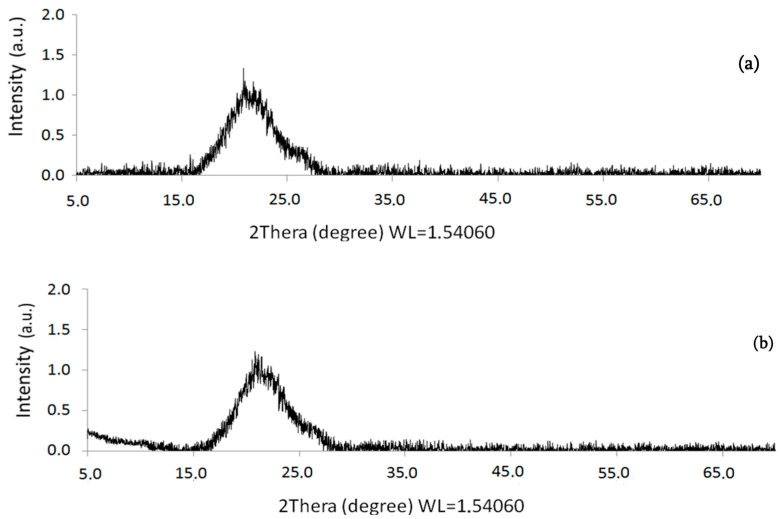
Powder X-ray diffraction XRD (PXRD) of the [I-Cu]_n_ (**a**) and [II-Cu]_n_ (**b**).

**Figure 9:**
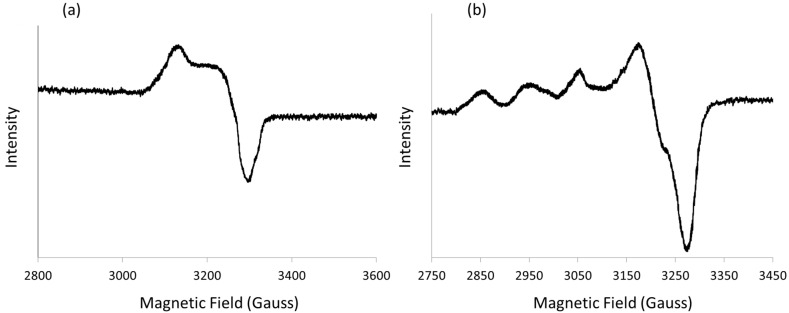
Powder Electron Paramagnetic Resonance (EPR) spectrum of Cu-[I-Cu]_6_ (**a**) and [I-Cu]_n_ (**b**).
